# Pharmaco-economic impact of demographic change on pharmaceutical expenses in Germany and France

**DOI:** 10.1186/1471-2458-12-894

**Published:** 2012-10-23

**Authors:** Wolfgang Boecking, Anna Klamar, Florian Kitzmann, Wilhelm Kirch

**Affiliations:** 1 Research Association PH S-SA, Dresden, Germany

**Keywords:** Pharmaceutical expenses, Demographic changes, Health care systems, Germany, France

## Abstract

**Background:**

Most European health care systems are suffering from the impact of demographic change. In short, aging of society is leading to higher costs of treatment per capita, while reproduction rates below 2.1 children per woman lead to a reduced number of younger people to provide for the necessary contributions into the health insurance system.

This research paper addresses the questions what impact the demographic development will have on one particular spending area, what are pharmaceutical expenditure in two of Europe’s largest health care systems, Germany and France, and what the implications are for pharmaceutical companies.

**Methods:**

The research is based on publicly available data from German and French health ministries, the OECD, and institutes which focus on projection of demographic development in those countries. In a first step, data was clustered into age groups, and average spending on pharmaceuticals was allocated to that. In the second step, these figures were extrapolated, based on the projected change in the demographic structure of the countries from 2004 until 2050. This leads to a deeper understanding of demand for pharmaceutical products in the future due to the demographic development as a single driving factor.

**Results:**

Pharmaceutical expenses per head (patient) will grow only slightly until 2050 (0.5% p.a. in both countries).

Demographic change alone only provides for a slowly growing market for pharmaceutical companies both in Germany and in France, but for a relevant change in the consumption mix of pharmaceutical products, based on a shift of relevance of different age groups.

**Conclusions:**

Despite demographic changes pharmaceutical expenses per head (patient) and the overall pharmaceutical markets will grow only slightly until 2050 in Germany as well as in France. Nevertheless, the aging of society implies different challenges for pharmaceutical companies and also for the health care system. Companies have to cope with the shift of relevance of different age groups and within the health care system new options for financing the slowly growing expenses have to be found.

## Background

According to the statistical projections held in 2003 by the German and the French national institutes of statistics (Statistisches Bundesamt and INSEE), both countries are expecting considerable aging of their populations over the next decades until 2050. This demographic change is inevitable although the forecast is more severe in Germany than in France. As a result of the persistent low fertility rates in Germany (less than 1.4 children per woman in 2003), the demographic structure of the population will change from a pyramid to a more balanced structure during the next decades. The current average ratio of 3:1 (people below 60 and people above 60) will change to 2:1 or even 1:1 during the following 30–50 years from 2004 according to the different scenarios of demographic aging [1-3].

In France, fertility rates are higher (1.9 children per woman in 2003) and the average fertility rate for the years between 1988 and 2003 has been 1.8 [4]. Therefore, the French population is also aging but the process is slower which means that the pyramid structure persists longer than in Germany.

The impact of demographic aging on social security, health care, pensions, education and unemployment is on the agenda of all European countries [5,6]. As medical consumption increases with age, the change in the demographic structure towards a larger group of older people suggests an increase of the medical costs [7-10].

This research paper analyzes the pharmaco-economic impact of demographic aging by taking into account only one medical spending area: pharmaceutical expenses. Isolating one cost-driving factor leads to a deeper understanding of how the demand for pharmaceutical products is likely to develop prospectively in two of Europe’s largest health care systems, Germany and France, based on the changes in the demographic structure and size of the population, and what the implications are for pharmaceutical companies. Hence, this research paper explicitly excludes other factors such as medical progress and a change in consumptive behavior for pharmaceutical products, but focuses solely on the impact of the change in demographic structures.

As pointed out before, Germany and France have relevant different demographic projections in terms of speed of the aging process and change of the size of population. A comparative analysis of these two countries helps to understand the impact of the demographic component on pharmaceutical expenditure in two different environments.

## Methods

This research is based on publicly available data from German and French health ministries, the OECD, and institutes which focus on the projection of demographic developments in those countries.

In both countries, demographic projections until 2050 were published in 2003 and so 2004 was set as the consistent reference year. In both projections different scenarios were proposed. The German scenario employed in this model has the following assumptions: a low mortality rate, a fertility rate of 1.4 and a net migration level of 200.000 per year [1]. This corresponds to the widely spread hypothesis that the fertility rate observed in the last 20 years before 2003 will maintain unchanged in the future, mortality will stay low as the average life expectancy has grown steadily during the last decades and migration has the tendency to be more important than today [11].

The French scenario (France without overseas colonies) runs under the assumptions of a low mortality rate, a fertility rate of 2.1 and a net migration level of 100.000 per year [4]. As the French fertility rate has been between 1.8 and 2.1 since 1980 [12,13], the authors assume that higher fertility will persist until 2050. The mortality rate is as low as in the German scenario, and the migration effect corresponds in France as well as in Germany to the highest projection made by the national institutes of statistics.

In the context of this paper pharmaceutical expenditure refers to turnovers with drugs that were prescribed by physicians in a considered period. Data on pharmaceutical expenses in Germany is based on an analysis of the overall pharmaceutical costs per age held in 2004 for the whole statutory health insurance system which covers 90% of the German population [14,15]. Data on pharmaceutical expenses in France relies on an analysis of the average pharmaceutical costs per head in 1997 of a representative group sample in the French social security system, which covers nearly 100% of the population [16,17]. As pharmaceutical expenses in France are higher than in Germany since 1970 in terms of price and volume [18-21], and since the focus of this research lies on analyzing the impact of the demographic structure over time, the slight difference of the reference date for the data (1997 for France and 2004 for Germany) is considered negligible in this model.

Overall the statistical analysis for this research paper is based on publicly available data which were used for calculations with Microsoft Excel. Data for the projected change in the demographic structure (2004–2050) and for average spending on pharmaceuticals in Germany and France (reference year) were provided by the already mentioned institutes or organizations. So predicted figures for changes in population and weights of age groups were given. Based on this, data for average spending on pharmaceuticals could be extrapolated by weighting data for the reference year and for 2050 with the corresponding weight of age groups in that year.

In a last step figures for 2004 and for 2050 were compared and a growth rate was calculated.

### Assumptions

The model is based on an analysis of the impact of demographic change, analyzing the demographic effect as the only driving factor for pharmaceutical expenses until 2050. All other influences on pharmaceutical spending as medical progress as well as behavioral aspects of drug consumption are not taken into account.

This approach enables us to analyze the impact of one single cost-driving factor and provides deeper insight into the sensitivity of data on this particular component. It also helps to clarify the notion of an aging society hanging over all public spending areas like a sword of Damocles as pointed out in many knowledgeable publications [5,22]. This means that it deliberately neglects other factors that might influence the development of pharmaceutical expenditure until 2050, and therefore does not reflect the true overall development of pharmaceutical expenses, but the effect of demographic change as one single cost-driving factor.

Step 1 Clustering data into age groups

In order to be able to allocate and extrapolate data for pharmaceutical expenses in Germany and France within comparable age groups, we clustered information into age groups of 10 years, which is based on the availability of the required information. Thus, nine clusters were created (0–9, 10–19, 20–29, 30–39, 40–49, 50–59, 60–69, 70–79 and above 79 years of age) which incorporate the total population – male and female – within the age group per country for the years 2004 and 2050 respectively.

The same approach was applied to the average pharmaceutical expenses per head. This enables the projection of expenses per age cluster over time, based purely on the change in the demographic structure of the countries.

Step 2 Projecting expenses

In the second step, the total population per country within the clusters is converted into the relative percentage of population within the respective cluster, in order to enable us to reflect the relative importance of that age group in 2004 as well as 2050. Pharmaceutical expenses, on the other hand, remain the same. This is done to isolate the effect of demographic change alone, under the assumption that we do not change the pharmaceutical “treatment” of the population within each age group. Therefore, this approach enables us to identify the true impact of demographic change alone on the development of pharmaceuticals, based on two different demographic developments.

Pharmaceutical expenses per age group are then multiplied with the relative weight of that respective age group in 2004 and 2050 respectively, in the purpose of understanding the change within each age group, as well as the total expenses in that year.

The data and the analysis for Germany are shown in detail in the Tables 1 and 2, for France in the Tables 3 and 4 respectively.

**Table 1:** Data and analysis Germany, part 1

**Alter**	**2004**	**2050**	**2004 in %**	**2050 in %**	**Pharma Expenses 2004**	**Partial Expenses 2004**	**Partial Expenses 2050**	**Change Expenses**
0-9	7694,90	5821,66	9,29%	7,63%	99,01 €	9,20 €	7,55 €	-17,93%
10-19	9141,19	6282,01	11,04%	8,23%	106,10 €	11,72 €	8,73 €	-25,45%
20-29	9725,63	7705,55	11,75%	10,10%	104,27 €	12,25 €	10,53 €	-14,06%
30-39	12309,02	8878,96	14,87%	11,63%	157,36 €	23,40 €	18,31 €	-21,75%
40-49	13324,47	9107,03	16,09%	11,93%	229,86 €	37,00 €	27,43 €	-25,86%
50-59	10085,68	9816,77	12,18%	12,86%	409,99 €	49,95 €	52,74 €	5,58%
60-69	10385,48	10330,74	12,54%	13,54%	525,72 €	65,95 €	71,16 €	7,90%
70-79	6602,22	8448,77	7,97%	11,07%	728,26 €	58,08 €	80,62 €	38,81%
>79	3519,48	9928,30	4,25%	13,01%	781,84 €	33,24 €	101,71 €	206,00%
SUM	82788,06	76319,81	100,00%	100,00%		300,77 €	378,78 €	25,93%

Source: Own analysis based on [1,14].

**Table 2:** Data and analysis Germany, part 2

	**2004**	**2050**
Ratio age 20-60 / >60	1,22	0,87
Change ratio in %	-28,50	
Change population in %	-7,81	
Total Pharmaexpenses in bn EUR	24,90	28,91
Change in total expenses	16,1	

Source: Own analysis based on [1,14].

**Table 3:** Data and analysis France, part 1

**Alter**	**2004**	**2050**	**2004 in %**	**2050 in %**	**Pharma Expenses 1997**	**Partial Expenses 2004**	**Partial Expenses 2050**	**Change Expenses**
0-9	7279,22	8658,88	12,15%	11,60%	210,44 €	25,58 €	24,41 €	-4,57%
10-19	7612,54	8513,59	12,71%	11,40%	143,91 €	18,29 €	16,41 €	-10,28%
20-29	7643,13	8306,88	12,76%	11,13%	222,42 €	28,38 €	24,75 €	-12,81%
30-39	8602,58	8583,24	14,36%	11,50%	310,69 €	44,63 €	35,72 €	-19,95%
40-49	8482,64	8176,95	14,16%	10,95%	411,76 €	58,32 €	45,10 €	-22,66%
50-59	7797,79	7787,49	13,02%	10,43%	770,93 €	100,37 €	80,42 €	-19,88%
60-69	5198,12	7813,66	8,68%	10,47%	1084,67 €	94,14 €	113,53 €	20,59%
70-79	4611,94	7219,29	7,70%	9,67%	1592,33 €	122,61 €	153,98 €	25,58%
>79	2665,04	9594,43	4,45%	12,85%	1613,83 €	71,81 €	207,41 €	188,83%
SUM	59893,00	74654,40	100,00%	100,00%		564,13 €	701,73 €	24,39%

Source: Own analysis based on [4,16].

**Table 4:** Data and analysis France, part 2

	**2004**	**2050**
Ratio age 20-60 / >60	1,19	0,79
Change ratio in %	-33,87	
Change population in %	24,65	
Total Pharmaexpenses in bn EUR	33,79	52,39
Change in total expenses	55,04	

Source: Own analysis based on [4,16].

## Results

**Figure 1:**
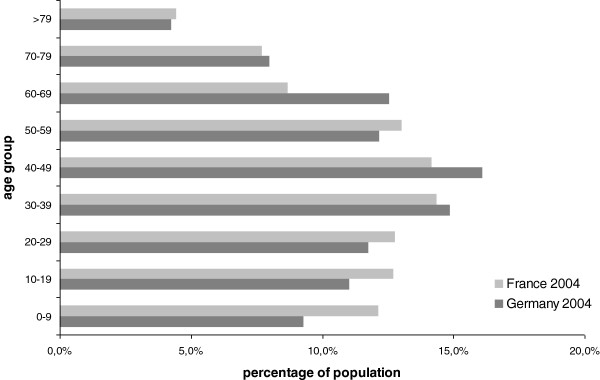
**Demographic structure of Germany and France in 2004**[1,4].

**Figure 2:**
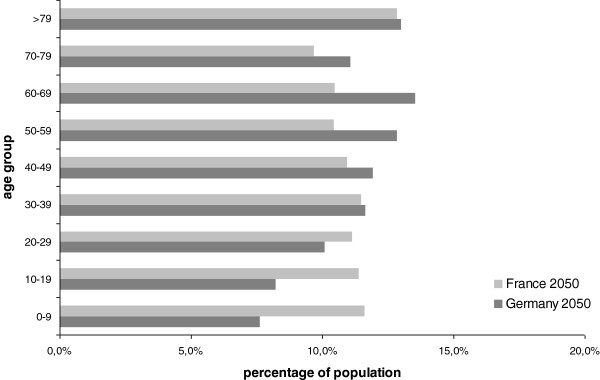
**Projected demographic structure of Germany and France in 2050**[1,4].

The analysis shows that the change in the demographic structure of both countries is not a relevant driver of costs of pharmaceutical expenses per capita. The projected annualized growth of per capita expenses – based on demographic change – is only 0.5% p.a., or approximately 25% from 2004 to 2050. The rate of change is almost identical for Germany and France, despite quite relevant differences in the projected demographic changes in both countries (i.e. fertility rates of 1.4 in Germany as compared to 2.1 in France). This is mostly due to the different levels of per capita expenses per age group in the two countries. Figures 1 and 2 illustrate the demographic structures of Germany and France in 2004 and 2050.

**Figure 3:**
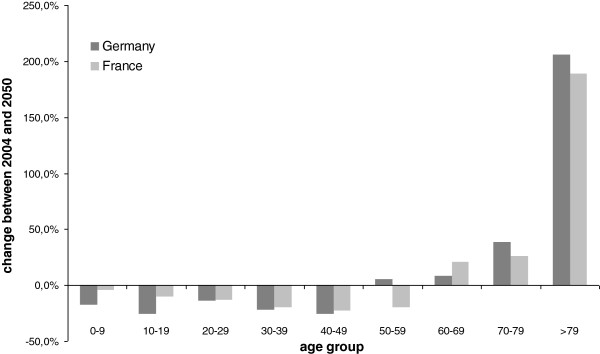
**Projected change in pharmaceutical expenses per age group in Germany and France between 2004 and 2050 **[14,16].

In detail, the results of the analysis are:

– The demographic effect on average pharmaceutical expenses (per head) from 2004 to 2050 is low in both countries. In Germany, the average per capita expenses increase by 26% over 46 years. In France, during the same period the increase is 24%. This represents an annual increase of only 0.5%.

– The aging of society leads to a shift of costs in both countries from the younger to the older people. The structural change has no essential impact on the average costs per head. In both countries, pharmaceutical expenses are declining in the younger segments and increasing in the older segments. According to the respective shift in population, Germany’s decline in the younger segments and increase in the older segments is more substantial than in France. This results in a significant change of relevance of different age groups for pharmaceutical expenses, as illustrated in Figure 3.

– Fertility rate is the only major differentiation factor when analyzing total pharmaceutical expenses instead of per capita expenses. The overall volume of pharmaceutical expenses in France grows stronger than in Germany in consequence of a projected increase in the population in France (60 Mio in 2004 and 75 Mio in 2050) as compared to a projected decrease in Germany (83 Mio in 2004 and 76 Mio in 2050). Overall pharmaceutical expenses in Germany will increase by 16%, as compared to 55% in France from 2004 to 2050 [Tables 2, 4].

– Both countries will encounter a challenge in financing these slowly growing expenses in the future, as the ratio working population to non-working population is decreasing due to an overall aging in both countries. Taking the ratio of “people between 20 and 59” versus “people below 20 and above 59 years of age” as an approximate indicator for the working-age population in these countries, this ratio decreases in Germany from 1.22 in 2004 to 0.87 in 2050 which corresponds to 29%, while in France the ratio decreases from 1.19 in 2004 to 0.79 in 2050 (34%) [Tables 2, 4].

## Discussion

This analysis shows that the impact of demographic change on per capita pharmaceutical expenses is low, with a growth of only about 0.5% p.a. from 2004 to 2050. At first glance, this contradicts the leading opinion that demographic change will lead to a substantial increase in health care expenditures [23,24]. What appears even more surprising is that the relevant differences in projected fertility rates for Germany and France, 1.4 and 2.1 respectively, do not appear to make any difference at all. Does this mean that demographic change will not lead to higher expenses in our health care systems, and that the relevance of fertility rates on the health care system is overrated?

The answer to that question is not as simple as the results of this study may indicate. With respect to per capita expenses for pharmaceuticals – in a scenario where only the demographic structure changes and all other components stay the same, especially pharmaceutical costs per head for each age group – the answer to the two questions above is yes. Demographic-induced expenses for pharmaceuticals per capita will grow only slightly, independent of the different fertility rate scenarios.

However, there are four additional aspects to consider: a) consumption / product mix changes, b) development of overall market size, c) financing, and d) changes in other relevant factors.

Ad a) Consumption / product mix changes

While overall expenses per capita do not change a lot under the analyzed scenario, the changes per age group do change quite significantly, as shown in graphic 3. In this graphic, it can be clearly seen that lower age groups lose weight (more in Germany than in France, as a result of a lower fertility rate), while higher age groups grow of significance. Since the pharmaceutical products used in those age groups typically differ, the overall consumption pattern will also change, placing a higher relevance and demand for products that are targeted more towards the elderly. For the pharmaceutical industry, this results in a changed product mix, and hence leads to a reallocation of research and development expenditures.

Ad b) Development of overall market size

While the per capita expenses change only slightly at an almost identical rate between the two countries, the overall markets develop differently. In Germany, a lower population (−7.8% in the projected scenario) leads to a market decrease, which is partly offset by the projected increase of 26% in per capita expenses, leading to a total increase of 16% over 46 years [Table 2]. This implies almost a stagnation of the overall market size, based on the demographic development alone. In France, a growing population of projected 25%, combined with slightly increased per capita expenses of 24%, lead to a projected market growth of 55% over 46 years [Table 4]. While the annual growth rate is low as well, from a macroeconomic perspective, the implications of demographic change let the French market appear more promising to providers of pharmaceutical solutions.

Ad c) Financing

Although expenses per head grow slightly, the percentage of contributors to beneficiaries in both health care systems decreases. This means that both countries will be encountering a challenge in financing these slowly growing expenses in the future. The ratio working population to non-working population is decreasing in both countries due to an overall aging in both countries. Taking the ratio of “people between 20 and 59” versus “people below 20 and above 59 years of age” as an approximate indicator for the working age-population in these countries, this ratio decreases in Germany from 1.22 in 2004 to 0.87 in 2050 which corresponds to a decrease of contributors of approximately 29%, while in France the ratio decreases from 1.19 in 2004 to 0.79 in 2050 (34%). This implies that either the cost of health insurance per head needs to increase from 2004 to 2050 to finance the implications of the change in the demographic structure [25,26] (even more so in France than in Germany), or that the providers of pharmaceuticals have to find additional payment opportunities outside the public health insurance systems, such as marketing their products more directly to the end-user within existing legal restrictions.

Ad d) Changes in other relevant factors

To isolate the impact of the change in the demographic structures on pharmaceutical expenses, this study assumed that other factors remain constant. The results of that analysis are stated above. In addition to the findings of this study, there are other relevant factors such as medical progress, a change of consumption patterns within each age group over time [27], new products for higher age groups, price caps for certain products, duration of patents for new products, the relevance of generic drugs, and many more that can have further impact on the overall development of pharmaceutical expenses [28-30]. In the past decades, these factors have led to growth rates that were significantly higher than those resulting from demographic change alone [23,31]. This implies that the development of pharmaceutical expenses is more dependent on those other factors, than on demographic change (fertility, mortality and migration rates).

With respect to demographic change alone, the pharmaco-economic implications are:

– In terms of market growth, France appears to be a more interesting market for pharmaceutical companies in the future than Germany,

– A shift in the medical needs: Pharmaceutical companies will have to base their research more and more on indications aiming to help diseases of the elderly to address the growing significance of those age groups with respect to their product offerings,

– Price pressure leads to a growing demand for efficiency and low-cost providers (generic drugs) and increases the relevance of marketing and sales within existing legal restrictions. Further, there appears to be a growing need to find income sources outside the public health care systems, such as out-of-pocket payments from the patient or additional private insurance [32].

Nevertheless, medical progress and behavioral factors may boost the demand for pharmaceuticals beyond the impact of demographic development alone.

### Limitations

All the discussed findings are based on two already mentioned limitations. Firstly the demographic effect is considered as the only driving factor for pharmaceutical expenses until 2050 and other effects are neglected. Secondly the reference year for the data is 2004. The reasons for using 2004 as the reference year were already described in the methods section.

## Conclusions

In summary, the implications are as follows:

– Pharmaceutical expenses per head (patient) will grow only slightly until 2050. The effect of demographic aging on per capita expenses, both in Germany and France, amounts to only about 0.5% p.a.

– Due to the specific consumption patterns for pharmaceutical expenses in both countries, the relevant differences in fertility rates between Germany and France do not affect the projected development of per capita expenses with respect to demographic change.

– The relevance of specific age groups changes over time, requiring pharmaceutical companies to adapt to a change in the mix of pharmaceutical products, based on the different consumption patterns of different age groups.

– Due to a population decline in Germany and an increase in France until 2050, overall pharmaceutical expenses will increase by 16% in Germany and by 55% in France. While growth for France appears significantly higher, one has to take into account that this covers a 46-year period. This means that demographic change alone provides only for a slowly growing market for pharmaceutical companies in France and in Germany.

– However, this demand is increasingly difficult to be financed under the current public health insurance systems in Germany and in France because the projected low growth in demographic-induced expenses is accompanied by an aging society with decreasing ratios of “contributors to beneficiaries”.

– Therefore, pharmaceutical companies will face a need to take a role as

∘ innovator and premium brand leader in indications for the elderly,

∘ low-cost provider for solutions in age groups with declining relevance, and/or

∘ finding a more direct access to private customers and private payments as the financial pressure on overall pharmaceutical expenditure persists due to insufficient financing possibilities of the public health care systems.

## Competing interests

The authors declare that they have no competing interests.

## Authors' contributions

WB prepared the conception, performed the statistical analysis and wrote the first draft. AK, WK and FK substantially contributed to the interpretation of data, drew conclusions and critically revised the manuscript. All authors read and approved the final manuscript.

## Pre-publication history

The pre-publication history for this paper can be accessed here:

http://www.biomedcentral.com/1471-2458/12/894/prepub
